# *TP53* mutations, expression and interaction networks in human cancers

**DOI:** 10.18632/oncotarget.13483

**Published:** 2016-11-21

**Authors:** Xiaosheng Wang, Qingrong Sun

**Affiliations:** ^1^ Department of Basic Medicine, School of Basic Medicine and Clinical Pharmacy, China Pharmaceutical University, Nanjing 211198, China; ^2^ School of Science, China Pharmaceutical University, Nanjing 211198, China

**Keywords:** TP53 mutations, TP53 expression, TP53 interaction networks, human cancers, synthetic lethality

## Abstract

Although the associations of p53 dysfunction, p53 interaction networks and oncogenesis have been widely explored, a systematic analysis of *TP53* mutations and its related interaction networks in various types of human cancers is lacking. Our study explored the associations of *TP53* mutations, gene expression, clinical outcomes, and *TP53* interaction networks across 33 cancer types using data from The Cancer Genome Atlas (TCGA). We show that *TP53* is the most frequently mutated gene in a number of cancers, and its mutations appear to be early events in cancer initiation. We identified genes potentially repressed by p53, and genes whose expression correlates significantly with *TP53* expression. These gene products may be especially important nodes in p53 interaction networks in human cancers. This study shows that while *TP53*-truncating mutations often result in decreased *TP53* expression, other non-truncating *TP53* mutations result in increased *TP53* expression in some cancers. Survival analyses in a number of cancers show that patients with *TP53* mutations are more likely to have worse prognoses than *TP53*-wildtype patients, and that elevated *TP53* expression often leads to poor clinical outcomes. We identified a set of candidate synthetic lethal (SL) genes for *TP53*, and validated some of these SL interactions using data from the Cancer Cell Line Project. These predicted SL genes are promising candidates for experimental validation and the development of personalized therapeutics for patients with *TP53*-mutated cancers.

## INTRODUCTION

*TP53* mutations and dysfunction occur in more than half of all human cancer cases [1], and are independent markers of poor prognoses in some cancers [2]. In addition to mutations in *TP53* itself, mutations in p53 pathway genes are significantly enriched in cancer [3]. Thus, the study of the p53 pathway and its interaction networks is a promising source of insight for discovering therapeutic targets for *TP53*-mutated cancers [4]. Although the associations of p53 dysfunction, p53 interaction networks, and oncogenesis have been widely explored [5], a systematic analysis of *TP53* mutations and the related interaction networks in various types of human cancers is lacking. The Cancer Genome Atlas (TCGA) datasets cover 33 different cancer types and more than 10,000 cancer cases in total (https://gdc-portal.nci.nih.gov/). Each TCGA cancer type contains different types of “omics” data, including: whole exome (genome) sequencing; genomic DNA copy number arrays; DNA methylation; mRNA expression array and RNA-Seq data; microRNA sequencing; reverse-phase protein arrays; and clinical metadata. There have been a number of studies of genomic alterations across cancer types based on TCGA data [6–8]. However, few of them have focused on systematically exploring genomic alterations of *TP53* and its related interaction networks across a number of different cancer types.

Some therapeutic strategies have been proposed to treat *TP53*-mutated cancers, such as restoring wild-type activity to; promoting the degradation of; or targeting pathways regulated by mutant p53 [9]. We have suggested a strategy of identifying synthetic lethality gene pairs involving *TP53* for the development of a treatment for *TP53*-mutated cancers [10]. Two genes are synthetic lethal (SL) if dysfunction of either alone does not result in cell death, but dysfunction of both does [11]. Thus, the targeted disruption of a gene that is SL for *TP53* should selectively kill cancer cells with somatic mutations in *TP53,* but spare normal *TP53*-wildtype cells. The large amount of cancer genomic data available in TCGA now enable us to identify potential SL genes for *TP53*.

In this study we explored genomic alterations of *TP53* and its interaction networks by analyzing TCGA data across 33 human cancer types. We analyzed *TP53* mutation and gene expression data to identify potential nodes in *TP53* interaction networks, and performed survival analyses based on *TP53* mutations and expression profiles across the 33 cancer types, respectively. We also identified potential SL genes for *TP53* to find molecular targets for personalized therapy of *TP53*-mutated cancer patients.

## RESULTS

### *TP53* mutations in cancer

We calculated *TP53* mutation rates for 33 cancer types (Table 1). Almost one-third of cancer types have a *TP53* mutation rate greater than 50%, and more than one-half have a rate greater than 30%. The two cancer types with the highest *TP53* mutation rates affect women: uterine carcino-sarcoma (UCS) (91.2%) and ovarian serous cystadeno-carcinoma (OV) (83%). The other eight cancer types with a *TP53* mutation rate that exceeds 50% include four gastro-intestinal cancers: esophageal carcinoma (ESCA), rectal adeno-carcinoma (READ), pancreatic adeno-carcinoma (PAAD) and colon adeno-carcinoma (COAD); two lung cancers: lung squamous-cell carcinoma (LUSC) and lung adeno-carcinoma (LUAD); and head-and-neck squamous-cell carcinoma (HNSC) and brain lower-grade glioma (LGG). For each cancer type, we ranked the affected genes in decreasing order of mutation rate (Supplementary Table S1). We found that *TP53* has the highest mutation rate in six cancer types: UCS, OV, ESCA, LUSC, HNSC and sarcoma (SARC), and the second-highest mutation rate in seven other cancer types: READ, LUAD, LGG, bladder urothelial carcinoma (BLCA), stomach adeno-carcinoma (STAD), liver hepato-cellular carcinoma (LIHC), and breast-invasive carcinoma (BRCA). If we exclude the extremely long *TTN* gene, which has the highest mutation rate in eight cancer types, we find that *TP53* has the highest mutation rate in ten cancer types, and is one of the top three genes with the highest mutation rate in 16 cancer types. These data confirm that *TP53* is frequently mutated in a wide variety of cancer types.

**Table 1:** Mutation rates of TP53 in the 33 TCGA cancer types

Cancer type	Full name	Mutation rate (%)^a^	Rank^b^
UCS	uterine carcino-sarcoma	91.2	1
OV	ovarian serous cystadeno-carcinoma	83	1
ESCA	esophageal carcinoma	82.7	1
LUSC	lung squamous-cell carcinoma	82	1
READ	rectum adeno-carcinoma	79	2
HNSC	head-and-neck squamous-cell carcinoma	71.6	1
PAAD	pancreatic adeno-carcinoma	64.7	3
COAD	colon adeno-carcinoma	56	3
LUAD	lung adeno-carcinoma	54.2	2
LGG	brain lower-grade glioma	53.5	2
BLCA	bladder urothelial carcinoma	49.9	2
STAD	stomach adeno-carcinoma	47.5	2
SARC	sarcoma	36.1	1
KICH	kidney chromophobe	33.3	6
LIHC	liver hepato-cellular carcinoma	31.1	2
BRCA	breast-invasive carcinoma	30.9	2
UCEC	uterine corpus endometrial carcinoma	28.6	7
GBM	glioblastoma multiforme	23.4	6
ACC	adrenocortical carcinoma	20	70
SKCM	skincutaneous melanoma	15.3	459
PRAD	prostate adeno-carcinoma	12	4
CHOL	cholangio-carcinoma	11.1	49
DLBC	lymphoid neoplasm diffuse large B-cell lymphoma	10.4	248
LAML	acute myeloid leukemia	8.1	8
CESC	cervical squamous-cell carcinoma and endocervical adeno-carcinoma	4.5	347
THYM	thymoma	3.3	182
KIRP	kidney renal papillary-cell carcinoma	2.5	170
KIRC	kidney renal clear-cell carcinoma	2.4	152
TGCT	testicular germ-cell tumors	1.4	3280
THCA	thyroid carcinoma	0.8	624
PCPG	pheochromocytoma and paraganglioma	0.6	2938
UVM	uveal melanoma	0	NA

a *TP53* mutation rates are listed in decreasing order.

b The rank of the TP53 mutation rate in the corresponding cancer type.

*TP53* has a relatively low mutation rate in some cancer types, such as thymoma (THYM) (3.3%); kidney renal papillary-cell carcinoma (KIRP) (2.5%); kidney renal clear-cell carcinoma (KIRC) (2.4%); testicular germ-cell tumors (TGCT) (1.4%); thyroid carcinoma (THCA) (0.8%); pheochromocytoma and paraganglioma (PCPG) (0.6%); and uveal melanoma (UVM) (0%). However, most of these cancers are relatively rare. Surprisingly, there are marked differences in the *TP53* mutation rates in cancers from the same organ but different cell types, e.g., in KIRP, KIRC, and kidney chromophobe (KICH), with rates of 2.5%, 2.4% and 33.3%, respectively.

**Figure 1:**
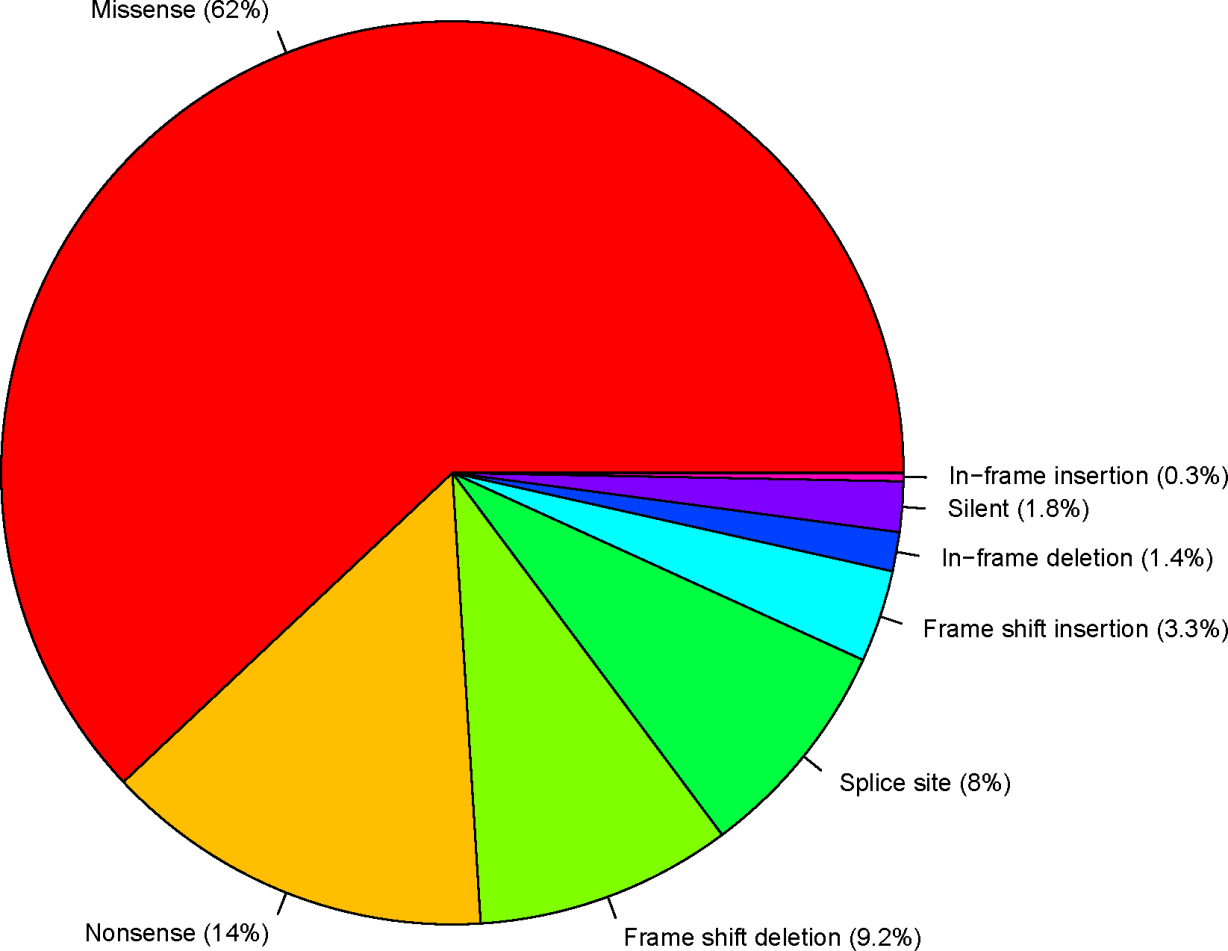
*TP53* variant classification

*TP53* mutations are comprised of eight classes: missense, nonsense, frame-shift deletion, frame-shift insertion, in-frame deletion, in-frame insertion, silent and splice-site. Figure 1 summarizes the proportion of each class of *TP53* mutations in all 33 TCGA cancer types. The most frequent classes of *TP53* mutations are missense (62%), nonsense (14%) and frame-shift deletions (9%). The proportions of each class of mutations in all of the *TP53* mutations in each cancer type are listed in Supplementary Table S2. In general, nonsense mutations, frame-shift deletions, frame-shift insertions and splice-site mutations are all highly deleterious, and 34.5% of all *TP53* mutations fall into one of these classes. In contrast, in-frame deletions, in-frame insertions, and silent mutations have comparatively much less deleterious effects, but only 3.5% of all *TP53* mutations fall into one of these mutation classes. Since most *TP53* missense mutations are deleterious [12], we can conclude that deleterious or altered-function mutations predominate among *TP53* mutations discovered in cancers.

### Genes with elevated expression levels in *TP53*-mutated cancers

#### Genes that are more highly expressed in *TP53*-mutated cancers compared to *TP53*-wildtype cancers

The most important function of p53 is to act as a transcription factor that directly or indirectly regulates thousands of other genes [13]. Upon exposure to stress stimuli such as DNA damage, hypoxia, or oncogene activation, p53 acts as a tumor suppressor by transcriptional repression of oncogenes [14]. Once mutations compromise p53′s transcriptional repression function, genes that are usually repressed by it should have elevated expression in *TP53*-mutated cancers compared to *TP53*-wildtype cancers or normal tissue. We identified genes potentially repressed by p53 by comparing gene expression levels in cancers with non-silent (functionally significant) *TP53* mutations compared to *TP53*-wildtype cancers in the TCGA datasets. Genes that are highly expressed in *TP53*-mutated cancers compared to *TP53*-wildtype cancers were identified in 29 cancer types (four cancer types were excluded from the analysis due to their small numbers of *TP53*-mutated samples) and are listed in Supplementary Table S3 (fold change > 1.5, false discovery rate (FDR) < 0.05). We call these loci “TP53-MW” genes.

There are 48 TP53-MW genes identified in at least 10 different cancer types (Supplementary Table S4). Two of these genes (*CDKN2A* and *KIF2C*) were identified in 13 different cancer types, and 10 genes (*DEPDC1*, *CENPF*, *CDC20*, *KIF14*, *CENPA*, *NUF2*, *ANLN*, *TTK*, *FAM72D* and *OIP5*) in 12 different cancer types. The expression level of *CDKN2A* (cyclin-dependent kinase inhibitor 2A) in *TP53*-mutated cancers is higher than in *TP53*-wildtype cancers in 13 different cancer types: BLCA, BRCA, LIHC, LUAD, PAAD, SARC, SKCM, UCEC, COAD, GBM, LGG, STAD, and THYM. The *CDKN2A* gene encodes two proteins: p16INK4a and p14ARF, which both act as tumor suppressors by regulating the cell cycle [15]. p14ARF has been reported to activate p53 by promoting degradation of the product of the *MDM2* proto-oncogene, which targets p53 [15]. Our results indicate that p53 may in turn inhibit *CDKN2A* such that loss of p53 function results in upregulation of *CDKN2A*. The expression level of another gene, *KIF2C* (kinesin family member 2C), is also higher in *TP53*-mutated cancers than in *TP53*-wildtype cancers in 13 different cancer types: BRCA, LIHC, LUAD, UCEC, KIRC, PAAD, ACC, PRAD, BLCA, SKCM, STAD, SARC and LGG. This gene encodes a kinesin-like protein that may regulate cellular senescence of human primary cells via a p53-dependent pathway [16]. A previous study has shown that *KIF2C* expression is significantly suppressed by p53 in breast cancer cells [17], a finding consistent with our results from the TCGA datasets (e.g., BRCA).

**Figure 2:**
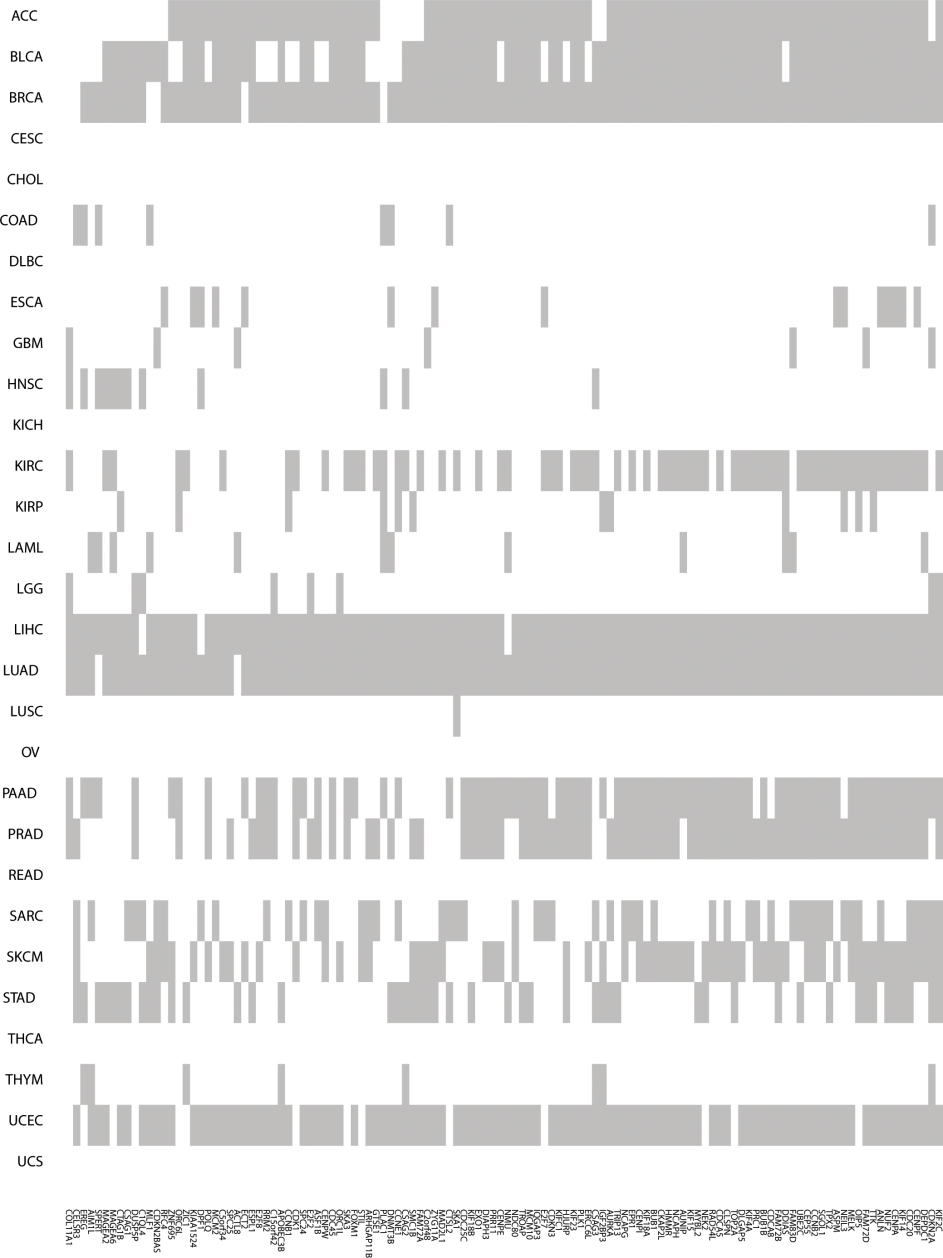
Higher-expression-level genes in *TP53*-mutated cancers compared to *TP53*-wildtype cancers The grey color indicates that a gene is more highly expressed in *TP53*-mutated cancers and the white color indicates that it isn't.

**Figure 3:**
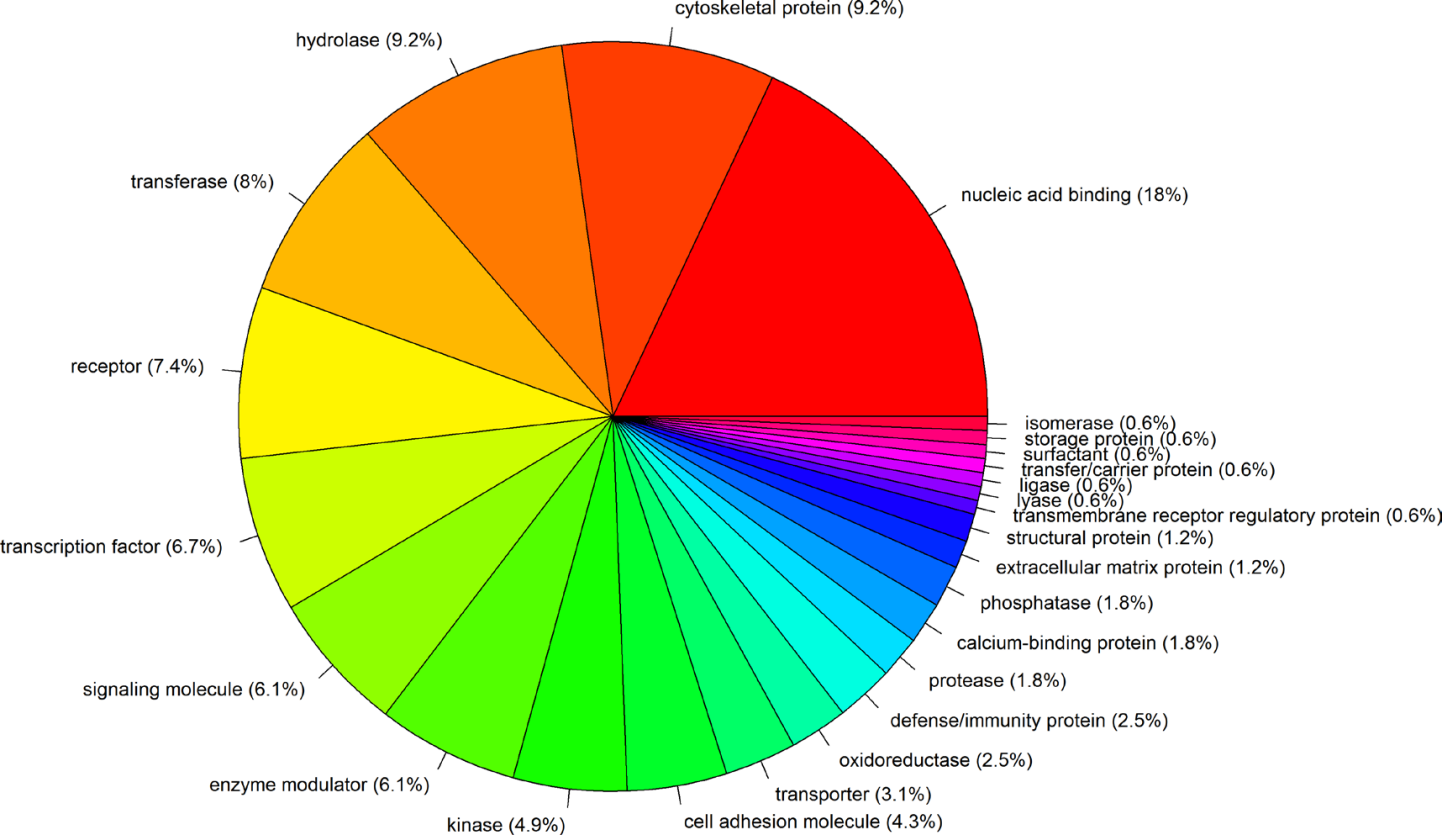
Protein classes of 120 genes that are more highly expressed in *TP53*-mutated cancers compared to *TP53*-wildtype cancers

**Figure 4:**
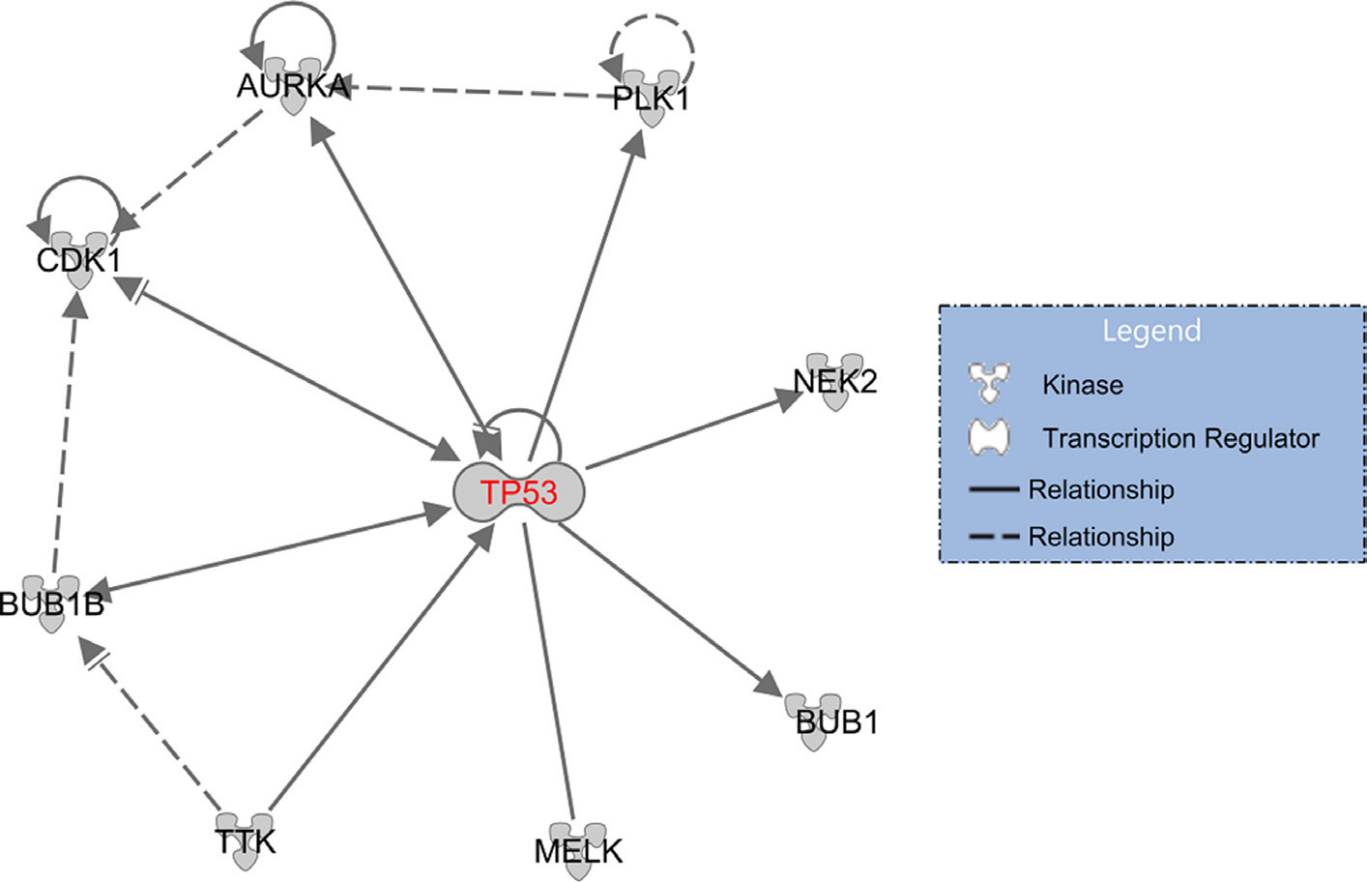
*TP53* regulates or interacts with eight kinase-encoding genes A solid line indicates a direct interaction, and a dashed line an indirect interaction; an arrow pointing from A to B indicates that A causes B to be activated, which includes any direct interaction, e.g., binding, phosphorylation, modification, etc.; an arrow ending with ““ pointing from A to B indicates that A causes B to be either activated or inhibited.

There are a total of 120 TP53-MW genes that are common to more than one-quarter of the 29 different cancer types (Figure 2 and Supplementary Table S3). These 120 genes encode 25 different classes of proteins (Figure 3). Of these, 18% encode nucleic-acid binding proteins, and the remainder encode various cytoskeletal proteins, hydrolases, transferases, receptors, transcription factors, signaling molecules, and enzyme modulators and kinases, indicating that p53 transcriptionally represses genes encoding many different classes of proteins. Eight members of this class of 120 TP53-MW genes encode protein kinases: *AURKA*, *BUB1*, *BUB1B*, *CDK1*, *MELK*, *NEK2*, *PLK1* and *TTK*. The products of these eight genes are of particular interest as targets for the development of small-molecule kinase inhibitors, a strategy adopted by several cancer therapies [18]. Since *TP53* mutations are not directly druggable, targeting druggable SL partners of *TP53* may be a promising approach to the treatment of *TP53*-mutated cancers [10]. Network analysis of the gene set made up of *TP53* and these eight kinase-encoding genes using IPA (Ingenuity Pathway Analysis) software shows that *TP53* directly interacts with all of these genes, confirming the relevance of these identified genes to p53 (Figure 4). In fact, previous studies have revealed that all the eight kinase-encoding genes interact with *TP53* [19–25].

Using the PANTHER Classification System [26], we categorized the 120 TP53-MW genes into eight molecular function classes: binding (GO:0005488), catalytic activity (GO:0003824), channel regulator activity (GO:0016247), enzyme regulator activity (GO:0030234), nucleic acid binding transcription factor activity (GO:0001071), receptor activity (GO:0004872), structural molecule activity (GO:0005198), and transporter activity (GO:0005215). Most of the TP53-MW gene products are involved in binding and catalytic activity (> 70%), and the others are involved in nucleic-acid binding, transcription factor, receptor, structural molecule, transporter, enzyme regulator, and channel regulator activity (Supplementary Figure S1). Using Gene Set Enrichment Analysis (GSEA) software [27], we identified 75 canonical pathways significantly associated with this 120-gene set, as shown in Supplementary Table S5 (FDR < 0.05). Supplementary Table S5 shows that these gene products are significantly involved in p53-related pathways such as cell cycle [28], p53 signaling, DNA replication [29], *PLK1* signaling [30], and Aurora A/B signaling [31].

### Genes that are more highly expressed in *TP53*-mutated cancers compared to *TP53*-wildtype cancers, and also higher in *TP53*-wildtype cancers than in normal controls

Among the genes that are more highly expressed in *TP53*-mutated cancers than in *TP53*-wildtype cancers, there is an interesting subset whose expression is also higher in *TP53*-wildtype cancers than in normal tissue. We call these loci “TP53-MWN” genes. In other words, a TP53-MWN gene's expression level follows this pattern: *TP53*-mutated cancers > *TP53*-wildtype cancers > normal controls. Thus a TP53-MWN gene has an elevated expression level in cancers relative to normal tissue, and further has an elevated expression in *TP53*-mutated cancers than in *TP53*-wildtype cancers, suggesting that TP53-MWN genes are oncogenic probably by the interaction of their expression products with p53.

To identify TP53-MWN genes, we first identified what we call “TP53-WN” genes, those with a higher expression level in *TP53*-wildtype cancers than in normal tissue (fold change > 1.5, FDR < 0.05) for 19 cancer types (14 cancer types were excluded from the analysis due to their small numbers or lack of normal samples). TP53-MWN genes are the intersection of the TP53-MW and TP53-WN gene sets (Supplementary Table S6). There are 130 TP53-MWN genes that are common to more than one-quarter of the 19 different cancer types (Supplementary Table S7). KEGG pathway analysis shows that these gene products are mostly involved in the cell cycle; the p53 signaling pathway; pathways in cancer and mismatch repair; and pathways that are specific for a number of different cancer types such as small-cell lung cancer, prostate cancer, bladder cancer, glioma, pancreatic cancer, melanoma, and chronic myeloid leukemia (FDR < 0.05; Supplementary Table S8).

**Figure 5:**
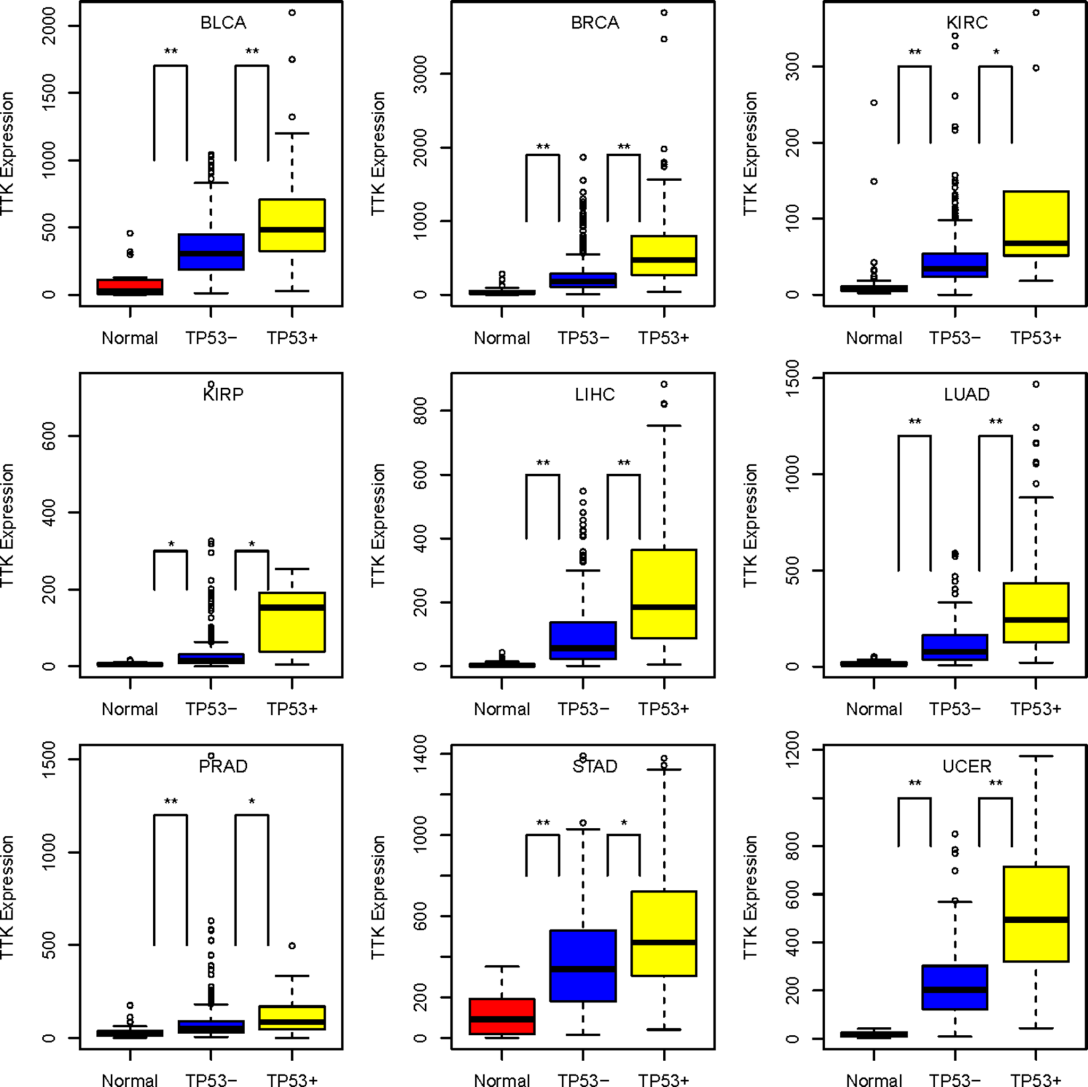
*TTK* gene expression level pattern: *TP53*-mutated cancers > *TP53*-wildtype cancers > normal controls, in nine cancer types *TP53*+: *TP53*-mutated cancers; *TP53*−: *TP53*-wildtype cancers; *10^−10^ < *P*-value ≤ 0.001; **P-value ≤ 10^−10^.

Of the 130 TP53-MWN genes, 11 encode protein kinases: *AURKA, AURKB, BUB1, BUB1B, CDK1, GSG2, MELK, NEK2, PLK1, PKMYT1* and *TTK*. In addition to the previously identified eight kinase-encoding TP53-MW genes, TP53-MWN genes include three more kinase genes: *AURKB*, *GSG2*, and *PKMYT1*, genes whose interactions with *TP53* have already been documented [32–34]. Of these 11 kinase genes, *TTK* follows the TP53-MWN expression pattern in nine different cancer types: BLCA, BRCA, KIRC, KIRP, LIHC, LUAD, PRAD, STAD, and UCEC (Figure 5). *TTK* encodes a serine/threonine kinase that has been implicated in the regulation of centrosome duplication and mitotic checkpoint response, and plays a role in the stabilization and activation of p53 during spindle disruption [23] (Figure 4). Our results indicate that p53 may in turn inhibit *TTK* by a negative feedback loop, since *TP53* mutations seem to cause the elevated expression of *TTK*.

### Genes that are more highly expressed in *TP53*-mutated cancers compared to normal tissue

We also identified genes that are more highly expressed in *TP53*-mutated cancers compared to normal tissue, but that are not more highly expressed in *TP53*-wildtype cancers compared to normal tissue. We call these loci “TP53-MSN” genes. We are interested in these genes because the mechanism underlying the elevated expression of TP53-MSN genes could be specifically related to *TP53* mutations. To identify TP53-MSN genes, we first identified genes with higher expression levels in *TP53*-mutated cancers than in normal tissue (fold change > 1.5, FDR < 0.05; we call these loci “TP53-MN” genes), and then identified genes with higher expression levels in *TP53*-wildtype cancers than in normal tissue (fold change > 1.2, FDR < 0.05; we call these loci “TP53-WN2” genes which obviously include the TP53-WN genes). TP53-MSN genes were obtained by subtraction of TP53-WN2 genes from the TP53-MN gene list (Supplementary Table S9). There are 27 TP53-MSN genes that are common to more than one-quarter of the 19 different cancer types (Supplementary Table S10). They encode a wide variety of proteins (Supplementary Figure S2), although more than 50% of them encode nucleic-acid binding proteins; transcription factors; and signaling or cell-adhesion molecules. Network analysis using STRING [35] shows that *TP53* inhibits *FOSL1*, *SPHK1*, *ICAM5*, and *MSLN*. In addition, our results suggest that *TP53* may also inhibit other TP53-MSN genes, and that *TP53* mutations may contribute to their elevated expression in a number of different cancer types.

### Correlation of *TP53* mutation rate with clinical phenotypes

We compared the *TP53* mutation rates among different clinical phenotypes of cancer patients including gender, race, tumor stage, size or direct extent of the primary tumor (T), lymph nodes (N), and metastasis (M) in 18 cancer types: ACC, BLCA, BRCA, CESC, CHOL, COAD, DLBC, ESCA, GBM, HNSC, KICH, KIRC, KIRP, LAML, LGG, LIHC, LUAD, and LUSC (Supplementary Table S11). We selected these clinical phenotypes and cancer types because there are relatively complete records of these clinical phenotypes in these 18 cancer types in the TCGA datasets. In DLBC the *TP53* mutation rate is lower in male than female subjects (unadjusted *P*-value = 0.05, odds ratio = 0), while in LIHC the rate is higher in male than female subjects (unadjusted *P*-value = 0.0007, odds ratio = 2.4). The other cancer types show no significant differences in *TP53* mutation rates between male and female subjects. For the race phenotype, only HNSC shows a significantly higher *TP53* mutation rate in African-American than in White-American subjects (unadjusted *P*-value = 0.03, odds ratio = 2.48). We did not find significant differences in the *TP53* mutation rates among different stages, T, N, or M status of tumor except that ACC shows a significantly higher *TP53* mutation rate in large-size cancers (T3, T4) than small-size cancers (T1, T2) (unadjusted *P*-value = 0.04, odds ratio = 3.42). Since the phenotypes tumor stage, T, N, and M reflect the development or progress status of tumors, our results indicate that *TP53* mutations are probably early events in tumorigenesis and drive its progression. This conclusion agrees with previous studies [36, 37].

### *TP53* mutations are associated with worse cancer outcome prognoses

**Figure 6:**
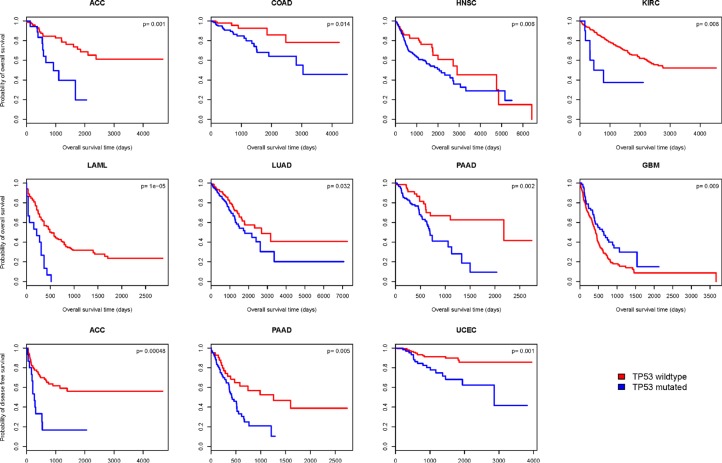
Kaplan–Meier survival curves show significant overall survival (OS) or disease-free survival (DFS) time differences between *TP53*-mutated and *TP53*-wildtype cancer patients (log-rank test, unadjusted *P*-value < 0.05)

We compared the overall survival (OS) time between *TP53*-mutated and *TP53*-wildtype cancers in 20 cancer types (13 cancer types were excluded from the analysis due to very few samples having both *TP53* mutation and survival data). Kaplan-Meier survival curves (Figure 6) show that patients with *TP53* mutations have significantly worse OS prognoses compared with those without *TP53* mutations in seven cancer types: ACC, COAD, HNSC, KIRC, LAML, LUAD, and PAAD; however, they have better OS prognoses in GBM (log-rank test, unadjusted *P*-value < 0.05). We also compared the disease-free survival (DFS) time between *TP53*-mutated and *TP53*-wildtype cancers in 18 cancer types (15 cancer types were excluded due to very few samples having both *TP53* mutation and recurrence data). Kaplan-Meier survival curves (Figure 6) show that patients with *TP53* mutations have significantly worse DFS prognoses compared with those without *TP53* mutations in three cancer types: ACC, PAAD, and UCEC (log-rank test, unadjusted *P*-value < 0.05). These results confirm that *TP53* mutations lead to poor clinical outcomes in a number of cancers [38–40].

### *TP53* gene expression in cancer

#### Identification of genes whose expression correlates with *TP53* expression in cancer

**Figure 7:**
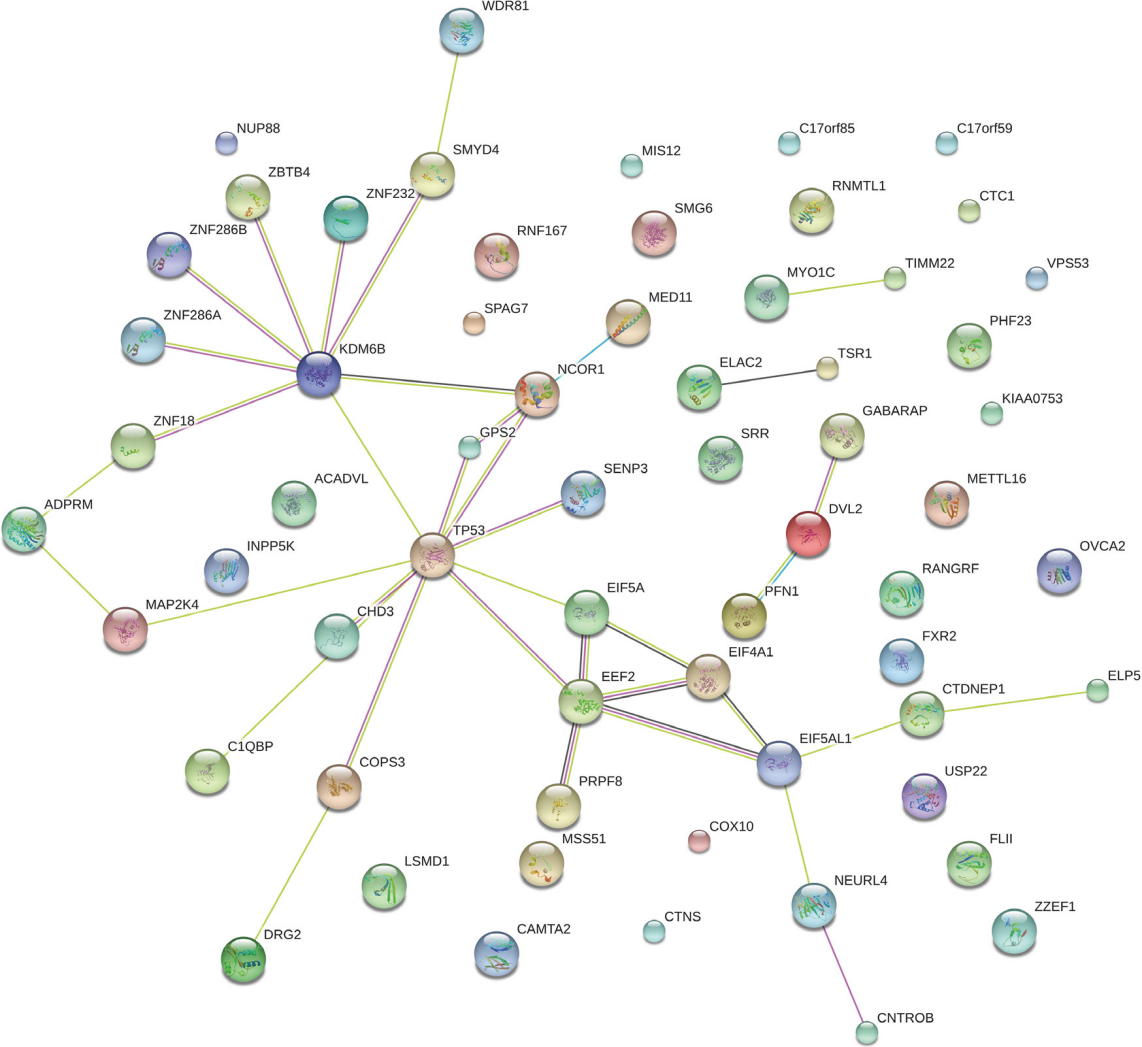
Network analysis of the 61 TP53-PCOR genes by STRING A TP53-PCOR locus is a gene whose expression positively correlates with *TP53* expression in cancers but not in normal tissue (Pearson product-moment correlation, FDR < 0.05).

We identified genes whose expression significantly correlates with *TP53* expression in 21 cancer types with normal controls (Pearson product-moment correlation, FDR < 0.05). Supplementary Tables S12 and S13 list genes whose expression positively or negatively correlates with *TP53* expression, respectively. Here we call loci whose expression levels positively or negatively correlate with *TP53* expression levels as “TP53-PCOR” or “TP53-NCOR” genes, respectively. There are 61 TP53-PCOR and 2 TP53-NCOR genes identified in at least 10 of the 21 cancer types examined (Supplementary Tables S12, S13). Network analysis of the 61 TP53-PCOR genes by STRING [35] shows that *TP53* directly interacts with *GPS2, NCOR1, SENP3, KDM6B, EIF5A, EEF2, MAP2K4, CHD3 and COPS3*, and indirectly interacts with a number of other genes via the hub nodes *KDM6B* and *EEF2* (Figure 7). Gene set enrichment analysis (GSEA) shows that 44 of the 61 TP53-PCOR genes are located in the cytogenetic band (chr17p13) where *TP53* is also located (FDR = 5.57*10^−80^), and that 9 others are located in cytogenetic band chr17p11 (FDR = 5.7*10^−9^). Thus, a total of 53 (or 87% of) TP53-PCOR genes are located in cytogenetic bands chr17p13,11 (Supplementary Table S14). In contrast, GSEA of 56 TP53-NCOR genes identified in at least one third of the 21 cancer types shows that except for four TP53-NCOR genes (*YPEL5, EPT1, LCLAT1*, and *CLIP4*) located in cytogenetic band chr2p23 (FDR = 0.003) and four (*MAGEA6, MAGEA12, MAGEA2*, and *CSAG1*) located in cytogenetic band chrxq28 (FDR = 0.015), no significant number of TP53-NCOR genes cluster in a cytogenetic band. This analysis reveals that in cancers, genes positively co-expressed with *TP53* mainly cluster near the *TP53* locus, while genes negatively co-expressed with *TP53* are sparsely distributed over the genome, and can be found at locations that are very distant from *TP53*.

### Differential expression of *TP53* among *TP53*-mutated cancers, *TP53*-wildtype cancers and normal tissue

We compared *TP53* expression among *TP53*-mutated cancers, *TP53*-wildtype cancers and normal tissue, and identified cancers with significantly different expression of *TP53* (*P*-value < 0.05). Table 2 shows that in 14 of 29 cancer types analyzed, *TP53* expression is significantly lower in *TP53*-mutated compared to *TP53*-wildtype cancers. It makes sense that some *TP53* mutations compromise p53 tumor suppressor function by reducing *TP53* expression. However, *TP53* expression is higher in *TP53*-mutated cancers than in normal tissue in some cancer types: GBM, KIRP and LUSC; whereas it is lower in some others such as LIHC, HNSC, KICH and PAAD. The explanation for these results could be that some *TP53* mutations result in overexpression of mutant forms of *TP53* while other mutations simply inactivate *TP53* [12]. In 19 TCGA cancer types with at least four normal control samples we found that *TP53* expression differed significantly between *TP53*-wildtype cancers and normal tissue in 11 cancer types. Interestingly, 10 of these 11 cancer types show elevated expression of *TP53* in their *TP53*-wildtype subtype compared to normal controls. These results confirm that *TP53* accumulation occurs in a number of cancers [41–43], and indicates that this accumulation is independent of *TP53* mutations.

**Table 2:** Comparison of TP53 expression

*TP53*-mutated vs. *TP53*-wildtype cancer		
Cancer type	*P*-value	Fold change^c^
SKCM	7.12*10^−22^	2.14
LIHC	1.08*10^−9^	1.64
HNSC	5.07*10^−8^	1.61
LUAD	6.95*10^−8^	1.34
BRCA	2.15*10^−6^	1.22
PRAD	3.32*10^−5^	1.32
UCEC	0.0002	1.35
STAD	0.0003	1.33
BLCA	0.001	1.27
THCA	0.002	1.46
DLBC	0.007	2.1
KIRC	0.017	1.24
ACC	0.02	1.37
COAD	0.045	1.23

*TP53*–mutated cancer vs. normal tissue		
Cancer type	*P*–value	Fold change^d^
GBM	3.23*10^−8^	5.7
LIHC	0.001	0.64
KIRP	0.002	1.75
HNSC	0.002	0.66
LUSC	0.007	1.41
KICH	0.03	0.75
PAAD	0.047	0.67

*TP53*–wildtype cancer vs. normal tissue		
Cancer type	*P*–value	Fold change^e^
KIRC	4.09*10^−27^	1.5
GBM	7.42*10^−18^	6.63
KIRP	1.31*10^−17^	1.83
THCA	2.64E*10^−15^	1.3
LUAD	4.22*10^−10^	1.52
CHOL	2.44*10^−6^	2.47
STAD	0.0004	1.54
COAD	0.0005	1.49
KICH	0.003	0.75
UCEC	0.004	1.53
PRAD	0.026	1.16

c Mean *TP53* expression in *TP53*-wildtype cancers / mean *TP53* expression in *TP53*-mutated cancers.

d Mean *TP53* expression in *TP53*-mutated cancers / mean *TP53* expression in normal tissue.

e Mean *TP53* expression in *TP53*-wildtype cancers / mean *TP53* expression in normal tissue.

We then divided *TP53* mutations into truncating and non-truncating classes to observe their respective effects on *TP53* expression. Truncating mutations include nonsense, frame-shift deletion, frame-shift insertion and splice-site, while non-truncating mutations include missense, in-frame deletion, and in-frame insertion (silent mutations were excluded from these analyses due to their minor effect on gene function). As expected, in 23 of 29 cancer types analyzed, *TP53* expression is significantly lower in *TP53*-truncated cancers than in other *TP53*-mutated cancers, and in the other six cancer types *TP53* expression does not significantly differ between them (*P*-value < 0.05, Supplementary Table S15). In 24 of the 29 cancer types, *TP53* expression is significantly lower in *TP53*-truncated cancers than in *TP53*-wildtype cancers, and in the other five cancer types *TP53* expression does not differ significantly between them (*P*-value < 0.05, Supplementary Table S16). In 14 of the 15 cancer types with both *TP53*-truncated cancers and normal samples to compare, *TP53* expression is significantly lower in *TP53*-truncated cancers than in normal tissue, and in one cancer type *TP53* expression does not differ significantly between them (*P*-value < 0.05, Supplementary Table S17). These results confirm that *TP53*-truncating mutations result in decreased *TP53* expression most likely by causing *TP53* mRNA decay [44, 45].

In contrast, *TP53* non-truncating mutations may result in increased *TP53* expression. Indeed, in nine of 31 cancer types analyzed, *TP53* expression is significantly higher in *TP53*-mutated but *TP53*-non-truncating cancers than in *TP53*-wildtype cancers, and only in one cancer type is *TP53* expression significantly lower in *TP53*-mutated but *TP53*-non-truncating cancers (*P*-value < 0.05, Supplementary Table S18). Also, in 11 of the 15 cancer types, *TP53* expression is significantly higher in *TP53*-mutated but *TP53*-non-truncating cancers than in normal tissue, and in the other four cancer types *TP53* expression does not differ significantly between them (*P*-value < 0.05, Supplementary Table S19). Interestingly, in eight cancer types (BRCA, LUSC, ESCA, SARC, OV, ACC, READ, and STAD), *TP53* expression follows this pattern: *TP53*-mutated but *TP53*-non-truncating cancers > *TP53*-wildtype cancers > *TP53*-truncated cancers. In 11 cancer types (BRCA, LUAD, LUSC, GBM, COAD, ESCA, BLCA, READ, PRAD, UCEC and STAD), *TP53* expression follows this pattern: *TP53*-mutated but *TP53*-non-truncating cancers > normal tissue > *TP53*-truncated cancers. Finally, in one cancer type (STAD), *TP53* expression follows this pattern: *TP53*-mutated but *TP53*-non-truncating cancers > *TP53*-wildtype cancers *>* normal tissue > *TP53*-truncated cancers.

### Association between *TP53* gene expression levels and cancer prognosis

**Figure 8:**
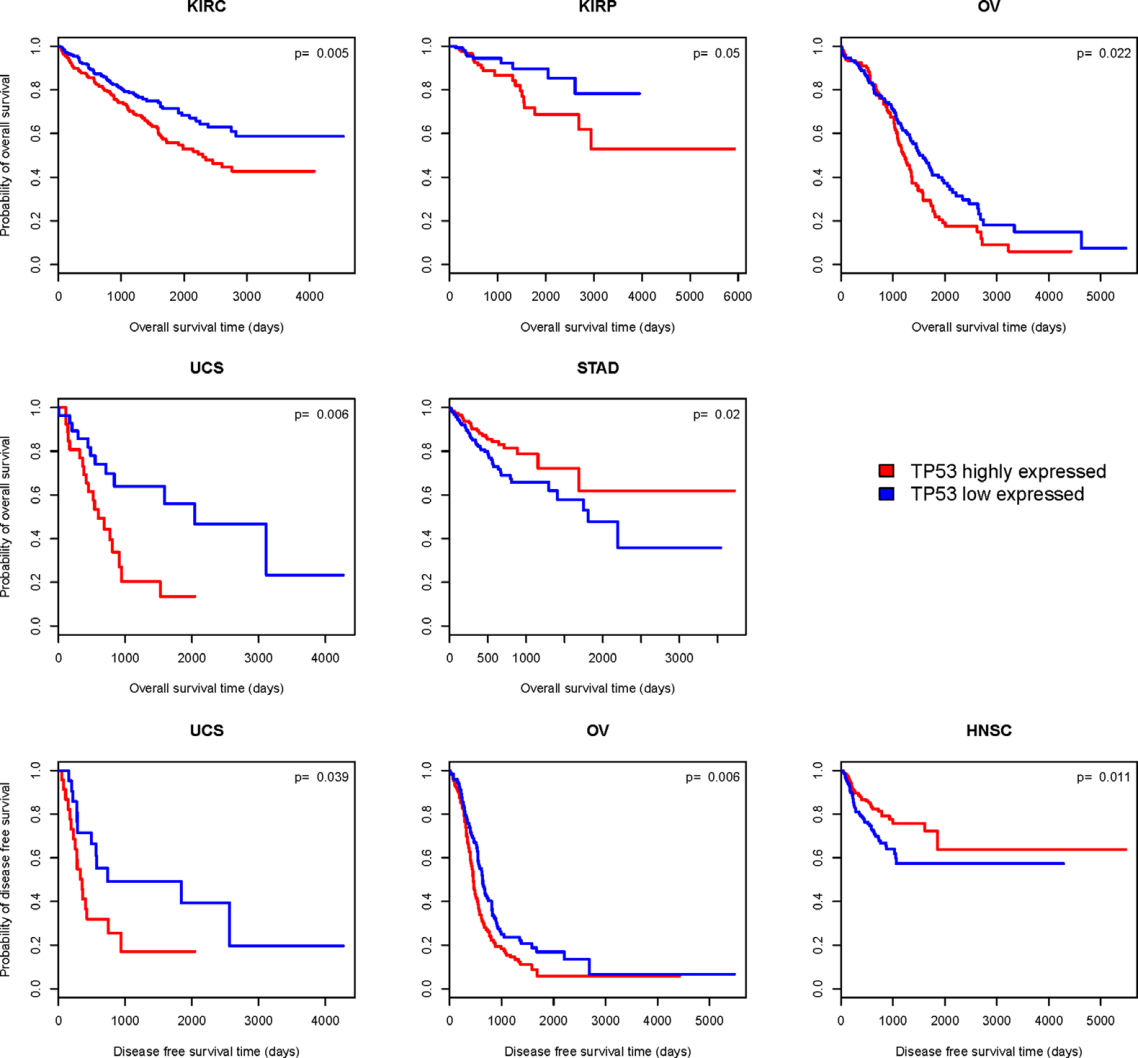
Kaplan–Meier survival curves show significant overall survival (OS) or disease-free survival (DFS) time differences between *TP53* higher-expression-level and *TP53* lower-expression-level cancer patients (log-rank test, unadjusted *P*-value < 0.05)

We compared OS and DFS between *TP53* higher-expression-level and *TP53* lower-expression-level cancers in 25 cancer types (eight cancer types were excluded from the analysis due to very few samples having both *TP53* expression and survival data). Kaplan-Meier survival curves (Figure 8) show that patients with higher expression levels of *TP53* have significantly worse OS prognoses than those with lower expression levels of *TP53* in four cancer types (KIRC, KIRP, OV and UCS), but have better OS prognoses in STAD (log-rank test, unadjusted *P*-value < 0.05). Patients with higher expression levels of *TP53* have significantly worse DFS prognoses than those with lower expression levels of *TP53* in two cancer types (UCS and OV), but have better DFS prognoses in HNSC (log-rank test, unadjusted *P*-value < 0.05). In the four cancer types (KIRC, KIRP, OV and UCS) in which higher expression levels of *TP53* are associated with significantly worse OS or DFS prognoses, *TP53* only shows significantly different expression in *TP53*-wildtype cancers (where it is higher) compared to *TP53*-mutated cancers in KIRK. Since *TP53*-mutated KIRCs have a worse OS prognosis than *TP53*-wildtype KIRCs (Figure 6), *TP53* lower-expression-level KIRCs should have a worse OS prognosis than *TP53* higher-expression-level KIRCs if the association between *TP53* expression and cancer prognosis correlates with *TP53* mutation status. Therefore, the association between *TP53* expression and cancer prognosis is unlikely to correlate with *TP53* mutation status in KIRC, KIRP, OV and UCS. However, in STAD and HNSC, where patients with higher expression levels of *TP53* have better OS or DFS prognoses than those with lower expression levels of *TP53* (Figure 8), the association could be confounded by *TP53* mutation status since *TP53* shows significantly higher expression levels in *TP53*-wildtype cancers compared to *TP53*-mutated cancers in STAD and HNSC (Table 2), and *TP53*-wildtype cancers have better OS or DFS prognoses than *TP53*-mutated cancers in a number of cancer types including HNSC (Figure 6). In fact, when we compared OS and DFS prognoses between *TP53* higher-expression-level and *TP53* lower-expression-level *TP53*-wildtype cancers, we found no significant association between *TP53* expression and cancer prognoses in either of these two cancer types. Thus, these results confirm that *TP53* accumulation leads to poor clinical outcomes in cancer. This is in line with the results of previous studies [42, 46].

We then examined the effects of both *TP53* mutation and expression status on patient survival (log-rank test, unadjusted *P*-value < 0.05). We found that *TP53*-mutated cancer patients with higher expression levels of *TP53* have significantly worse OS prognoses than *TP53*-mutated cancer patients with lower expression levels of *TP53* in ACC and UCS, and that the former category also have significantly worse DFS prognoses than the latter category in UCS. We also found that *TP53*-wildtype cancer patients with higher expression levels of *TP53* also have significantly worse OS prognoses than *TP53*-wildtype cancer patients with lower expression levels of *TP53* in KIRC, and that the former category also have significantly worse DFS prognoses than the latter category in BRCA. It was only in GBM that *TP53*-wildtype cancer patients with higher expression levels of *TP53* have significantly better DFS prognoses than *TP53*-wildtype cancer patients with lower expression levels of *TP53*. These results again indicate that elevated *TP53* expression is frequently associated with poor clinical outcomes in cancer.

### Identify potential SL genes for *TP53*

The identification of druggable SL gene partners for *TP53* may be an important approach to the treatment of *TP53*-mutated cancers, as mutant p53 is not directly druggable. To identify potential SL genes for *TP53* we hypothesize that *TP53* SL partners (oncogenes) are hyper-activated in *TP53*-mutated cancers compared to both *TP53*-wildtype cancers and normal tissue, as *TP53*-mutated cancer cells have to rely more heavily on *TP53*'s SL partners for survival than *TP53*-wildtype cancer cells or normal cells. We first examined the intersection of the TP53-MW and TP53-MSN gene lists for each of the relevant 19 cancer types described earlier (Supplementary Table S20; 14 cancer types were excluded from the analysis due to their small numbers or lack of normal samples). There are 1,863 such genes identified in at least one cancer type. Among the 1,863 potential SL genes for *TP53*, we are particularly interested in kinase-encoding genes because kinase inhibitors have been intensively investigated, and are a key class of anticancer drugs in clinical use or trials [18]. Table 3 lists 43 kinase-encoding genes in the 1,863 gene list. These kinase genes are mainly involved in p53 and cancer-related pathways: e.g., pathways in cancer, cell cycle, ERK signaling, MAPK signaling, TGF-β signaling, EGFR signaling, ErbB signaling, RAS signaling, axon guidance, focal adhesion, and the immune system. Table 3 shows that most of these kinases have clinically approved or investigational inhibitors or agonists. Among these kinase-encoding genes, *CDK6* (cyclin-dependent kinase 6) has been validated as an SL partner with *TP53* in a previous study [47]; and *EPHB2 MST1R*, *NEK2*, *PAK6*, *PLK1*, *SPHK1*, *STK31*, *STYK1* and *UCK2* have been predicted to be SL with *TP53* in a computational biology study [10].

**Table 3:** The 43 kinase-encoding genes that are potentially synthetic lethal for *TP53*

Symbol	Description	Cancer type^f^	Pathway^g^	Compound^h^
*ACVR1C*	activin A receptor, type IC	ESCA	TGF-β signaling	Adenosine triphosphate
*ALK*	anaplastic lymphoma receptor tyrosine kinase	UCEC	ERK signaling, MAPK signaling	Crizotinib, Crenolanib
*ALPK2*	alpha-kinase 2	UCEC	NA	NA
*BRDT*	bromodomain, testis-specific	HNSC, LIHC	NA	Xd14
*BRSK2*	BR serine/threonine kinase 2	STAD	LKB1 signaling	ADP
*CAMKV*	CaM kinase-like vesicle-associated	BRCA, STAD, UCEC	EGFR signaling	NA
*CDK6*	cyclin-dependent kinase 6	HNSC	Cell cycle, Pathways in cancer	Flavopiridol, Palbociclib
*CIT*	citron (rho-interacting, serine/threonine kinase 21)	ESCA, LIHC	ERK signaling	Cisplatin, Gemcitabine
*DAPK2*	death-associated protein kinase 2	KIRC	Pathways in cancer, Apoptosis	ADP
*EGFR*	epidermal growth factor receptor	HNSC	EGFR signaling, ErbB signaling, RAS signaling	Erlotinib, Gefitinib
*EIF2AK2*	eukaryotic translation initiation factor 2-alpha kinase 2	UCEC	Viral carcinogenesis, Immune system, EIF2 signaling	Adenosine triphosphate
*EPHA2*	EPH receptor A2	PAAD	Ras signaling, PI3K-AKT signaling	Dasatinib, Regorafenib
*EPHA8*	EPH receptor A8	UCEC	ERK signaling, Axon guidance	Adenosine triphosphate
*EPHB2*	EPH receptor B2	UCEC	ERK signaling, Axon guidance	Db04395
*EPHB3*	EPH receptor B3	BRCA	ERK signaling, Axon guidance	Adenosine triphosphate
*ERN2*	endoplasmic reticulum to nucleus signaling 2	PAAD	NA	Adenosine diphosphate
*FGFR3*	fibroblast growth factor receptor 3	STAD, UCEC	Ras signaling, Pathways in cancer, AKT signaling	Masitinib, Ponatinib
*GUCY2C*	guanylate cyclase 2C (heat stable enterotoxin receptor)	STAD	Metabolism, Pregnenolone biosynthesis	Linaclotide
*HUNK*	hormonally up-regulated Neu-associated kinase	COAD	NA	Adenosine triphosphate
*KIAA1804*	mixed lineage kinase 4	UCEC	NA	NA
*KSR2*	kinase suppressor of ras 2	LIHC, UCEC	Ras signaling, Immune system	Diglycerides Group
*LCK*	lymphocyte-specific protein tyrosine kinase	BRCA	Immune system, Ras pathway	Dasatinib, Ponatinib
*MAP4K1*	mitogen-activated protein kinase kinase kinase kinase 1	UCEC	MAPK signaling, TGF-β signaling	NA
*MAPK12*	mitogen-activated protein kinase 12	HNSC	MAPK signaling, TGF-β signaling, VEGF signaling, p53 signaling	Doramapimod
*MAPK15*	mitogen-activated protein kinase 15	PRAD	MAPK pathway, mTOR pathway, TNF signaling	NA
*MAST1*	microtubule associated serine/threonine kinase 1	UCEC	NA	Adenosine triphosphate
*MET*	met proto-oncogene (hepatocyte growth factor receptor)	LUAD, PAAD	Ras signaling, Axon guidance, Pathways in cancer, AKT signaling	Crizotinib, Foretinib, Tivantinib
*MST1R*	macrophage stimulating 1 receptor (c-met-related tyrosine kinase)	PAAD	ERK signaling, AKT signaling, MAPK signaling	Foretinib
*MYLK2*	myosin light chain kinase 2	KIRC	Focal adhesion, Oxytocin signaling	Prenylamine
*MYO3B*	myosin IIIB	HNSC	ERK signaling, PAK pathway	Adenosine triphosphate
*NEK2*	NIMA (never in mitosis gene a)-related kinase 2	PAAD	Cell cycle, Regulation of PLK1	Adenosine triphosphate
*NEK3*	NIMA (never in mitosis gene a)-related kinase 3	COAD	Prolactin signaling	Adenosine triphosphate
*PAK6*	p21 protein (Cdc42/Rac)-activated kinase 6	STAD	ErbB signaling, Ras signaling, Axon guidance, Focal adhesion	Guanosine triphosphate
*PKN1*	protein kinase N1	UCEC	cAMP signaling, ErbB signaling, Ras signaling, Axon guidance	Quercetin
*PLK1*	polo-like kinase 1	PAAD	Cell cycle, FoxO signaling	Gsk461364, Volasertib
*PTK6*	PTK6 protein tyrosine kinase 6	PAAD	Cell cycle, Signaling by ERBB2	Vandetanib
*RET*	ret proto-oncogene	HNSC	Pathways in cancer, Immune system	Sorafenib, Sunitinib, Imatinib, Vandetanib
*ROS1*	c-ros oncogene 1, receptor tyrosine kinase	BLCA	ERK signaling, AKT signaling, MAPK signaling	Crizotinib, Ceritinib
*STK31*	serine/threonine kinase 31	LIHC	Sperm motility	NA
*STYK1*	serine/threonine/tyrosine kinase 1	PAAD	Focal adhesion	Adenosine triphosphate
*TTBK1*	tau tubulin kinase 1	UCEC	NA	Adenosine triphosphate
*TYRO3*	TYRO3 protein tyrosine kinase	LIHC	ERK signaling, AKT signaling, MAPK signaling	BMS-777607
*WNK3*	WNK lysine deficient protein kinase 3	LUAD	Ion channel transport	Adenosine triphosphate

f Cancer types in which the kinase gene has a potential synthetic lethality relationship with *TP53*.

g Pathways the kinase gene is related to.

h Compounds are kinase inhibitors or agonists that have been approved or under investigation currently.

* Data on Pathways and Compounds are from the GeneCards (www.genecards.org), KEGG (www.genome.jp/kegg/), REACTOME (www.reactome.org/), TARGET (www.broadinstitute.org/cancer/cga/target), and DGIdb (dgidb.genome.wustl.edu) databases.

To further validate these potential *TP53* SL partners, we examined data from the Cancer Cell Line Project (http://www.cancerrxgene.org/). In a recent study [48], Iorio et al. identified a number of molecular markers of drug sensitivity using 265 compounds and pharmacogenomic screens in cancer cell lines. They found that *TP53*-mutated HNSCs are sensitive to the Rac GTPases inhibitor EHT 1864. Among the potential *TP53* SL partners in HNSC (Supplementary Table S20), we found that *PARVB* and *ARHGEF25* are associated with the activity of Rac GTPases. This demonstrates that *PARVB* and *ARHGEF25* have synthetic lethality relationships with *TP53* in HNSC. In addition, Iorio et al. found that *TP53*-mutated cancers are sensitive to the compounds Doxorubicin, Gemcitabine, Paclitaxel, Etoposide and 5-Fluorouracil. The targets of these compounds have been predicted to be *TP53*'s SL partners in this analysis, e.g., the genes *CYP2D6*, *NQO1*, *XDH*, *ABCC1*, *ABCC2*, *ABCC6*, *SLC22A16*, *CIT*, *SLC28A3*, *MAP4*, *UGT1A1*, *DPYD* and *UPP1* (Supplementary Table S20).

Moreover, we compared IC50 (drug concentration that reduces viability by 50%) values between *TP53*-mutated and *TP53*-wildtype cancer cell lines for each of the 265 compounds mentioned above. We found that BI-2536, GW843682X, Epothilone B, Afatinib and Gefitinib have significantly lower IC50 values in *TP53*-mutated cancer cell lines than in *TP53*-wildtype cancer cell lines (*P*-value < 0.05, FDR < 0.2, Supplementary Table S21). This indicates that *TP53*-mutated cancer cell lines are more sensitive to these compounds than *TP53*-wildtype cancer cell lines. The higher sensitivity of *TP53*-mutated cancer cell lines to these compounds could be attributed to the SL interactions between the targets of these compounds and *TP53*. Actually, the compounds' targets such as *PLK1* and *EGFR* have been identified to be SL with *TP53* by this method. Table 4 lists potential *TP53*'s SL partners that have supporting evidence provided by the Cancer Cell Line Project.

**Table 4:** The synthetic lethal genes with TP53 evidenced by the cancer cell line project

Symbol	Description	Compound^i^	Target pathway^j^
*PARVB*	parvin, beta	EHT 1864	cytoskeleton
*ARHGEF25*	Rho guanine nucleotide exchange factor (GEF) 25	EHT 1864	cytoskeleton
*CYP2D6*	cytochrome P450, family 2, subfamily D, polypeptide 6	Doxorubicin	DNA replication
*NQO1*	NAD(P)H dehydrogenase, quinone 1	Doxorubicin	DNA replication
*XDH*	xanthine dehydrogenase	Doxorubicin	LKB1 signaling
*ABCC1*	ATP-binding cassette, sub-family C (CFTR/MRP), member 1	Doxorubicin, Etoposide, Paclitaxel	DNA replication, cytoskeleton
*ABCC2*	ATP-binding cassette, sub-family C (CFTR/MRP), member 2	Doxorubicin, Etoposide, Paclitaxel	DNA replication, cytoskeleton
*ABCC6*	ATP-binding cassette, sub-family C (CFTR/MRP), member 6	Doxorubicin, Etoposide	DNA replication
*SLC22A16*	solute carrier family 22 (organic cation/carnitine transporter), member 16	Doxorubicin	DNA replication
*CIT*	citron rho-interacting serine/threonine kinase	Gemcitabine	DNA replication
*SLC28A3*	solute carrier family 28 (concentrative nucleoside transporter), member 3	Gemcitabine	DNA replication
*MAP4*	microtubule-associated protein 4	Paclitaxel	cytoskeleton
*UGT1A1*	UDP glucuronosyltransferase 1 family, polypeptide A1	Etoposide	DNA replication
*DPYD*	dihydropyrimidine dehydrogenase	5-Fluorouracil	DNA replication
*UPP1*	uridine phosphorylase 1	5-Fluorouracil	DNA replication
*PLK1*	polo-like kinase 1	BI-2536, GW843682X	mitosis
*EGFR*	epidermal growth factor receptor	Afatinib, Gefitinib	EGFR signaling
*MAST1*	microtubule associated serine/threonine kinase 1	Epothilone B	cytoskeleton
*TUBA4A*	tubulin, alpha 4a	Epothilone B	cytoskeleton
*TUBA8*	tubulin, alpha 8	Epothilone B	cytoskeleton

i Compounds and their targets data are from the Cancer Cell Line Project (www.cancerrxgene.org/) and DrugBank (www.drugbank.ca/).

j The target pathway data are from the Cancer Cell Line Project (www.cancerrxgene.org/).

## DISCUSSION

In our study we performed extensive analyses of *TP53* mutation, gene expression, and clinical data from 33 TCGA cancer type-specific datasets. We identified potential *TP53* interaction networks, the association between patient survival and *TP53* mutation or gene expression status, and potential druggable SL partners of *TP53*.

When comparing *TP53* expression levels among *TP53*-mutated (truncating mutations and non-truncating mutations) cancers, *TP53*-wildtype cancers and normal tissue, we found that *TP53* expression is consistently lower in *TP53*-truncated cancers compared to other cancers or normal tissue, while *TP53* expression is often higher in *TP53*-mutated but *TP53*-non-truncating cancers compared to other cancers or normal tissue. This indicates that *TP53*-truncating mutations reduce *TP53* expression but some non-truncating mutations are capable of increasing it. However, we did not find significant survival time (OS or DFS) differences between *TP53*-truncated and *TP53*-mutated but *TP53*-non-truncated classes of cancer patients, indicating that both types of *TP53* mutations are equally deleterious and lead to poor clinical outcomes. Interestingly, *TP53* expression is almost always elevated in *TP53*-wildtype cancers compared to normal controls in a number of cancer types. This indicates that p53 often accumulates in cancers, and that this accumulation could be stimulated by stress responses in cancer cells regardless of *TP53* mutation status. Furthermore, survival analyses suggest that p53 accumulation leads to poor clinical outcomes in many types of cancers.

Druggable SL gene partners for *TP53* may yield insights into the personalized treatment of patients with *TP53*-mutated cancers, as p53 mutants are not directly druggable. A successful example of applying the synthetic lethality approach is the targeting of cancers with dysfunction of the breast-cancer susceptibility genes 1 and 2 (*BRCA1* and *BRCA2*) by poly(adenosine diphosphate [ADP]–ribose) polymerase (*PARP*) inhibitors [49]. In the present study, we identified potential *TP53* SL partners based on the hypothesis that they should be overexpressed in *TP53*-mutated cancers compared to both *TP53*-wildtype cancers and normal tissue. Moreover, we validated some of the SL interactions by exploring the pharmacogenomic data from the Cancer Cell Line Project. One interesting finding is that there are five compounds to which *TP53*-mutated cancer cell lines are more sensitive than *TP53*-wildtype cancer cell lines, whereas there are many more (73) compounds to which *TP53*-wildtype cancer cell lines are more sensitive than *TP53*-mutated cancer cell lines (Supplementary Table S21). This implies that *TP53*-mutated cancers may have fewer treatment options and their proliferation is more difficult to control than *TP53*-wildtype cancers. Druggable *TP53* SL partners that are kinase-encoding genes are of particular interest. We identified 43 kinase-encoding genes that are potentially SL to *TP53*. Although these SL interactions need to be validated by experimental investigation, they represent a promising direction for future studies.

## MATERIALS AND METHODS

### Materials

We downloaded RNA-Seq gene expression data (Level 3), gene somatic mutation data (Level 2), and clinical data for all of the 33 cancer types for which data are available from the TCGA data portal (https://gdc-portal.nci.nih.gov/). For survival analyses we used clinical data from FireBrowse (http://gdac.broadinstitute.org/). We obtained pharmacogenomic data from the Cancer Cell Line Project (http://www.cancerrxgene.org/). The pharmacogenomic data cover 265 screened compounds and their targets, and include cancer cell lines' drug response, drug sensitivity, and somatic mutation data.

### Class comparison to identify differentially-expressed genes

We normalized TCGA RNA-Seq gene expression data by base-2 log transformation. We identified differentially expressed genes between two classes of samples using Student's *t* test. To adjust for multiple tests, we calculated adjusted *P*-values (FDR) for *t* test *P*-values. The FDR was estimated using the method of Benjami and Hochberg [50]. We used the threshold of FDR < 0.05 and mean gene-expression fold-change > 1.5 to identify the differentially expressed genes. In comparisons of *TP53* expression, we used the threshold of *P*-value < 0.05.

### Comparison of the *TP53* mutation rates among different clinical phenotypes

We compared the *TP53* mutation rates among different clinical phenotypes of cancer patients using Fisher's Exact Test. Each phenotype was divided into two classes: gender (male vs. female); race (African-American vs. White-American); tumor stage (early stage (Stage I-II) vs. late stage (Stage III-IV)); tumor size (T) (small size (T1-2) vs. large size (T3-4)); lymph nodes (N) (without regional lymph nodes (N0) vs. with lymph nodes (N1-3); metastasis (M) (no metastasis (M0) vs. metastasis (M1)). A threshold of *P*-value < 0.05 was used to evaluate the significance of differences in the *TP53* mutation rates between two classes of phenotypes.

### Expression correlation analysis

We identified genes whose expression significantly correlates with *TP53* expression in cancers, but does not correlate with *TP53* expression in normal tissue, by Pearson product-moment correlation analysis. Again, the FDR was used to adjust the *P*-value by the method of Benjami and Hochberg [50]. A threshold of FDR < 0.05 was used to evaluate the significance of expression correlations.

### Gene-set enrichment analysis

We categorized the sets of genes we identified into different molecular function classes or protein classes by the PANTHER Classification System [26]. We performed pathway analysis of the gene sets using KEGG (www.genome.jp/kegg/), REACTOME (www.reactome.org/) and the GSEA tool (http://software.broadinstitute.org/gsea/msigdb/). We carried out network analysis of gene sets using the Ingenuity Pathway Analysis tool (IPA, Ingenuity^®^ Systems, www.ingenuity.com) and STRING [35].

### Survival analyses

We performed survival analyses of TCGA patients based on *TP53* mutation data and *TP53* gene expression data, respectively. Kaplan-Meier survival curves were used to show the survival (overall survival or disease-free survival) differences between *TP53*-mutated cancer patients and *TP53*-wildtype cancer patients, and between *TP53* higher-expression-level patients and *TP53* lower-expression-level patients. *TP53* higher-expression-level and lower-expression-level patients were determined by the median values of *TP53* expression. If the *TP53* expression level in a patient was higher than the median value, the patient was classified as *TP53* higher-expression-level; otherwise as *TP53* lower-expression-level. We used the log-rank test to calculate the significance of survival-time differences between two classes of patients with a threshold of *P*-value < 0.05.

### Identification of potential SL genes for *TP53*

We first identified the set of genes whose expression is significantly higher in *TP53*-mutated cancers than in *TP53*-wildtype cancers (Student's *t* test, FDR < 0.05, fold change > 1.5), and then identified the set of genes whose expression is significantly higher in *TP53*-mutated cancers than in normal tissue (Student's *t* test, FDR < 0.05, fold change > 1.5), but not significantly higher in *TP53*-wildtype cancers than in normal tissue (Student's *t* test, FDR < 0.05, fold change > 1.2). We identified potential SL genes for *TP53* from the intersection of these two gene sets. To identify genes whose elevated expression is specifically related to TP53-mutated cancers, we believe that it is necessary to exclude as many genes as possible whose expression is significantly higher in TP53-wildtype cancers than in normal tissue. Therefore, we used a lower fold-change threshold of > 1.2 instead of > 1.5.

### Comparison of drug sensitivity between *TP53*-mutated and *TP53*-wildtype cancer cell lines

We compared IC50 values between *TP53*-mutated and *TP53*-wildtype cancer cell lines for compounds using Student's *t* test. We identified the compounds to which *TP53*-mutated and *TP53*-wildtype cancer cell lines have significantly different IC50 values using a threshold of *P*-value < 0.05.

## SUPPLEMENTARY MATERIALS




















