# Supplementary material for: TP53 mutations, expression and interaction networks in human cancers
Source: Oncotarget. 2016 Nov 21;8(1):624–43. doi: 10.18632/oncotarget.13483 (PMC5352183; doi:10.18632/oncotarget.13483)
Supplement: Supplementary file 1 [file oncotarget-08-624-s001.pdf]

TP53 mutations, expression and interaction networks in human cancers

Supplementary Materials

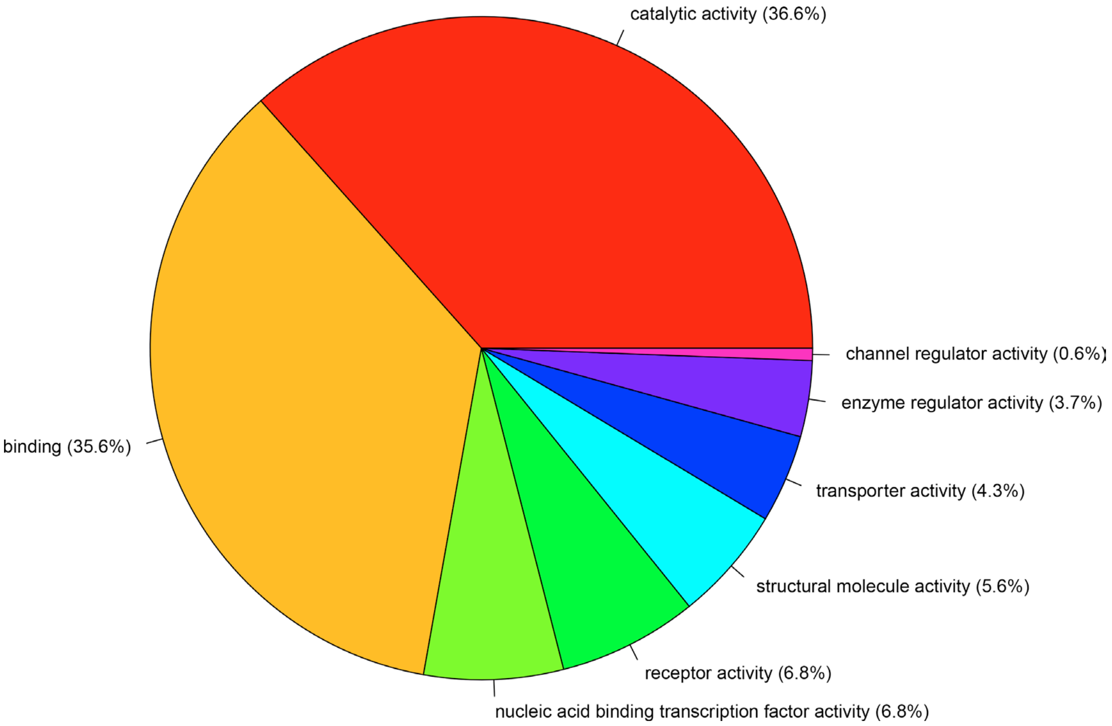

Supplementary Figure S1: Molecular function classification of the 120 genes that are more highly expressed in *TP53*-mutated cancers than *TP53*-wildtype cancers.

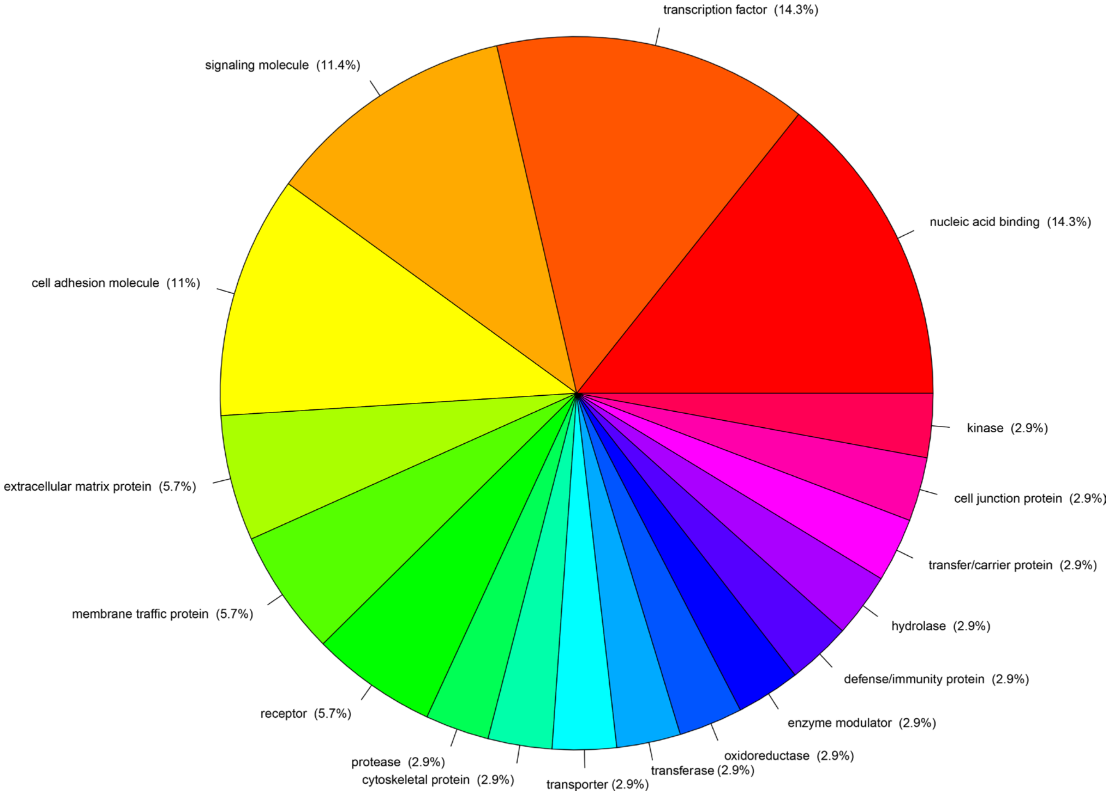

Supplementary Figure S2: Protein classes of the 27 TP53-MSN genes.

Supplementary Table S1: Gene mutation rates in cancers. See Supplementary_Table_S1

Supplementary Table S2: Proportions of each class of mutations for all of the *TP53* mutations in cancers. See Supplementary_Table_S2

Supplementary Table S3: Genes that are more highly expressed in *TP53*-mutated cancers than *TP53*-wildtype cancers. See Supplementary_Table_S3

Supplementary Table S4: The 48 genes that are more highly expressed in *TP53*-mutated cancers than *TP53*-wildtype cancers, and that are common to at least 10 of the different cancer types in which they are present. See Supplementary_Table_S4

Supplementary Table S5: Pathways that are significantly associated with the 120 TP53-MW gene set. See Supplementary_Table_S5

Supplementary Table S6: The genes whose expression follows the pattern: *TP53*-mutated cancers > *TP53*-wildtype cancers > normal controls. See Supplementary_Table_S6

Supplementary Table S7: The 130 genes whose expression follows the pattern: *TP53*-mutated cancers > *TP53*-wildtype cancers > normal controls; and that are common to more than one-quarter of the 19 different cancer types in which they are present. See Supplementary_Table_S7

Supplementary Table S8: KEGG pathways that are significantly associated with the 130 *TP53*-MWN gene set. See Supplementary_Table_S8

Supplementary Table S9: The genes whose expression is elevated in *TP53*-mutated cancers compared to normal tissue, but not elevated in *TP53*-wildtype cancers compared to normal tissue. See Supplementary_Table_S9

Supplementary Table S10: The 27 TP53-MSN genes common to at least five of the different cancer typesin which they are present. See Supplementary_Table_S10

Supplementary Table S11: Comparison of the *TP53* mutation rates among different clinical phenotypes of cancers. See Supplementary_Table_S11

Supplementary Table S12: Genes whose expression positively correlates with *TP53* expression in cancers. See Supplementary_Table_S12

Supplementary Table S13: Genes whose expression negatively correlates with *TP53* expression in cancers. See Supplementary_Table_S13

Supplementary Table S14: The 53 genes whose expression positively correlates with *TP53* expression, and which are located in cytogenetic bands chr17p13, 11.
See Supplementary_Table_S14

Supplementary Table S15: Comparison of *TP53* expression in *TP53*-truncated cancers versus *TP53*-mutated but *TP53*-non-truncated cancers

Cancer	*P*-value	Log2 (fold change)
**BRCA**	1.36E-68	−1.94
**HNSC**	3.22E-50	−1.61
**LUAD**	9.33E-41	−1.37
**STAD**	6.54E-33	−1.84
**LUSC**	1.37E-30	−2.13
**BLCA**	9.07E-28	−1.67
**ESCA**	4.55E-22	−1.74
**OV**	4.56E-22	−2.33
**COAD**	1.75E-20	−1.84
**LIHC**	1.10E-16	−1.86
**READ**	2.09E-14	−2.2
**SKCM**	1.16E-13	−1.8
**PAAD**	1.42E-13	−0.74
**PRAD**	2.64E-13	−1.42
**SARC**	1.26E-12	−1.5
**UCEC**	1.63E-11	−2.05
**LGG**	3.58E-07	−0.76
**KIRC**	0.0003	−1.33
**ACC**	0.0004	−1.94
**UCS**	0.001500328	−1.01
**GBM**	0.0049	−0.75
**CHOL**	0.0368	−2.81
**KICH**	0.0439	−0.61
DLBC	0.1199	−2.56
THYM	0.1334	−2.35
CESC	0.2076	−0.85
KIRP	0.3304	1.17
THCA	0.4981	−0.93
LAML	0.8492	−0.16

fold change = mean TP53 expression in TP53-truncated cancers / mean TP53 expression in TP53-mutated but TP53-non-truncated cancers.

The cancer types in which TP53 expression is significantly lower in TP53-truncated compared to TP53-mutated but TP53-non-truncated cancers (Student's *t* test *P*-value < 0.05) is highlighted in bold.

Supplementary Table S16: Comparison of *TP53* expression in *TP53*-truncated cancers versus *TP53*-wildtype cancers

Cancer	*P*-value	Log2 (fold change)
**BRCA**	3.04E-75	−1.45
**SKCM**	1.80E-39	−2.08
**LUAD**	4.93E-35	−1.25
**LIHC**	2.72E-28	−1.87
**HNSC**	1.11E-27	−1.56
**BLCA**	6.18E-23	−1.49
**STAD**	2.58E-22	−1.64
**UCEC**	1.02E-20	−2.16
**PRAD**	5.12E-16	−1.43
**COAD**	2.78E-12	−1.74
**ACC**	8.85E-09	−1.26
**KIRC**	5.43E-07	−1.42
**DLBC**	6.73E-06	−2.61
**LUSC**	3.93E-05	−0.96
**THCA**	0.0002	−0.78
**CHOL**	0.0002	−2.87
**READ**	0.0005	−1.58
**ESCA**	0.0006	−0.88
**PAAD**	0.0006	−0.54
**GBM**	0.0006	−0.76
**LGG**	0.0014	−0.61
**OV**	0.0071	−1.14
**SARC**	0.0272	−0.62
**THYM**	0.0351	−1.55
KIRP	0.074	0.94
KICH	0.1339	−0.36
LAML	0.5219	−0.22
CESC	0.6285	−0.3
UCS	0.7303	−0.29

fold change = mean TP53 expression in TP53-truncated cancers / mean TP53 expression in TP53-wildtype cancers.
The cancer types in which TP53 expression is significantly lower in TP53-truncated compared to TP53-wildtype cancers (Student's *t* test *P*-value < 0.05) is highlighted in bold.

Supplementary Table S17: Comparison of *TP53* expression in *TP53*-truncated cancers versus normal tissue

Cancer	*P*-value	Log2 (fold change)
**BRCA**	6.70E-32	−1.35
**LIHC**	1.32E-16	−1.79
**HNSC**	1.38E-15	−1.48
**LUSC**	1.72E-13	−0.95
**PRAD**	6.78E-12	−1.21
**COAD**	3.53E-09	−1.16
**LUAD**	1.48E-07	−0.64
**STAD**	3.48E-07	−1.02
**BLCA**	1.20E-05	−1.17
**UCEC**	0.0003	−1.54
**PAAD**	0.0009	−1.05
**GBM**	0.0019	1.97
**KICH**	0.0024	−0.78
**READ**	0.0031	−1.19
ESCA	0.1477	−0.47

fold change = mean TP53 expression in TP53-truncated cancers / mean TP53 expression in normal tissue.
The cancer types in which TP53 expression is significantly lower in TP53-truncated cancers compared to normal tissue (Student's *t* test *P*-value < 0.05) is highlighted in bold.

Supplementary Table S18: Comparison of *TP53* expression in *TP53*-mutated but *TP53*-non-truncated cancers versus *TP53*-wildtype cancers

Cancer	*P*-value	Log2 (fold change)
**BRCA**	2.14E-18	0.5
**LUSC**	9.15E-09	1.17
**ESCA**	3.26E-05	0.85
**SARC**	0.0002	0.88
**OV**	0.0003	1.19
**ACC**	0.0024	0.68
**READ**	0.0163	0.62
SKCM	0.0213	−0.28
**CESC**	0.0318	0.55
**STAD**	0.0472	0.2
BLCA	0.0556	0.19
LUAD	0.0574	0.13
THYM	0.0616	0.8
UCS	0.0697	0.71
PAAD	0.0757	0.2
LGG	0.1396	0.15
KICH	0.14	0.25
KIRP	0.2869	−0.24
UCEC	0.3217	−0.11
COAD	0.4142	0.1
TGCT	0.5547	−0.21
KIRC	0.5731	−0.08
HNSC	0.6206	0.05
THCA	0.6618	0.15
LAML	0.7309	−0.07
CHOL	0.8863	−0.06
PCPG	0.8967	−0.08
DLBC	0.9002	−0.05
PRAD	0.9521	−0.006
LIHC	0.9697	−0.004
GBM	0.9914	−0.001

fold change = mean TP53 expression in TP53-mutated but TP53-non-truncated cancers / mean TP53 expression in TP53-wildtype cancers.

The cancer types in which TP53 expression is significantly higher in TP53-mutated but TP53-non-truncated compared to TP53-wildtype cancers (Student's *t* test *P*-value < 0.05) is highlighted in bold.

Supplementary Table S19: Comparison of *TP53* expression in *TP53*-mutated but *TP53*-non-truncated cancers versus normal tissue

Cancer	*P*-value	Log2 (fold change)
**LUAD**	1.77E-19	0.73
**LUSC**	9.24E-18	1.18
**BRCA**	2.12E-15	0.6
**STAD**	4.72E-13	0.83
**GBM**	3.50E-12	2.73
**COAD**	1.93E-09	0.68
**ESCA**	7.64E-06	1.27
**READ**	3.62E-05	1.01
**BLCA**	0.0085	0.51
**PRAD**	0.0173	0.21
**UCEC**	0.035	0.51
PAAD	0.1045	–0.32
KICH	0.2142	–0.17
HNSC	0.2648	0.13
LIHC	0.5337	0.08

fold change = mean TP53 expression in TP53-mutated but TP53-non-truncated cancers / mean TP53 expression in normal tissue.

The cancer types in which TP53 expression is significantly higher in TP53-mutated but TP53-non-truncated cancers compared to normal tissue (Student's *t* test *P*-value < 0.05) is highlighted in bold.

Supplementary Table S20: Potential synthetic lethal (SL) genes for *TP53* in cancers.
See Supplementary_Table_S20

Supplementary Table S21: Comparison of IC50 values between *TP53*-mutated and *TP53*-wildtype cancer cell lines for 265 compounds. See Supplementary_Table_S21
